# The Healthy Hearts program to improve primary care for hypertension in seven rural health units of Iloilo Province, Philippines: a comparative cost study

**DOI:** 10.1186/s12875-025-02758-5

**Published:** 2025-03-22

**Authors:** Hilton Y. Lam, Haidee A. Valverde, Doris Mugrditchian, Muhammad Jami Husain, Soumava Basu, Bishal Belbase, Rauell John Santos, Dofel Joseph Calla, Tyrone Aquino, Andrew E. Moran, Deliana Kostova

**Affiliations:** 1 Institute of Health Policy and Development Studies, National Institutes of Health, University of the Philippines Manila, Manila, Philippines; 2 Resolve to save Lives, New York, USA; 3 Division of Global Health Protection, Centers for Disease Control and Prevention (CDC), Atlanta, GA USA; 4 World Health Organization, Manila, Philippines; 5 Columbia University Irving Medical Center, New York, USA

**Keywords:** Hypertension, Program costs, Philippines

## Abstract

**Background:**

In 2021, the Philippines launched the Healthy Hearts demonstration project for delivering hypertension (HTN) services in seven Rural Health Units (RHUs) in District 1 of Iloilo Province, West Visayas Region. This study evaluates the provider time cost and medication cost of delivering these services under three medication procurement scenarios, projecting them to the district and province levels to inform scaling-up efforts.

**Methods:**

A mixed-methods design was used for cost data collection, including key informant interviews (KII), focus group discussions (FGD), and secondary data sources. The HEARTS costing tool was adapted to analyze program costs per patient from the health system perspective. Three scenarios were assessed, depending on the procurement scheme of HTN medications: baseline local government procurement, pooled procurement through the Philippine Pharma Procurement Inc. (PPPI) national pooling mechanism, and private pharmacy outsourcing. We assessed annual provider labor costs and medication costs per patient for each scenario.

**Results:**

The average provider cost per patient was considerably lower for patients with controlled HTN than for patients with uncontrolled HTN: USD 5 (range USD 3.4–6.1 across RHUs) vs. USD 32.9 (range USD 28.8–38.4)) due to the need for more frequent follow-up visits for the latter. Average medication costs per patient were estimated at USD 9.1 (range USD 7.2–11.5) using local procurement prices, USD 2.9 (range USD 2.3–3.7) using PPPI pooled procurement prices, and USD 23 (range USD 17.9–30.5) using private pharmacy outsourced prices. The higher medicine costs in the pharmacy outsourcing scenario were partially offset by lower provider costs (an average reduction of USD 1.5 per patient per year) due to reduced on-site dispensing time in this scenario.

**Conclusions:**

The findings from this study indicate two key opportunities for cost savings in HTN management in the Philippines' rural health units system: 1) enhancing the control of HTN, thereby reducing the need for follow-up visits and cutting down on provider time costs, and 2) utilizing pooled medication procurement mechanisms such as through the Philippine Pharma Procurement Inc. Provider time costs can also be partially reduced through outsourcing the dispensing of medications to private pharmacies, although doing so is currently associated with higher medication costs, further underscoring the utility of pooled procurement mechanisms for essential hypertension medicines.

**Supplementary Information:**

The online version contains supplementary material available at 10.1186/s12875-025-02758-5.

## Contributions to the literature

### Cost analysis

Examines hypertension service costs in Philippine rural health units under three alternative strategies for medication procurement.

### Cost-saving opportunities

Identifies potential savings through improved hypertension control and pooled medication procurement.

### Importance of pooled procurement

Highlights the role of pooled medication procurement mechanisms for cost efficiency.

### Methodological insight

Utilizes mixed-methods approach for cost data collection and context-specific program costing model, offering practical guidance for healthcare policymakers and program managers.

## Background

Cardiovascular diseases (CVDs) cause more than one-third of all fatalities each year in the Philippines making it the leading cause of death in the country [1]. According to the 2018–2020 Expanded National Nutrition Survey, the prevalence of hypertension, the leading risk factor for CVD, was 20.9% among Filipinos 20 years and above [2]. In 2013, an estimated 65% of Filipinos with hypertension were aware of their condition, 37% were on treatment, and only 13% reached target blood pressure [3].

In 2018, the Healthy Hearts program was launched to address this public health concern. The overall strategy was to develop and scale up actions to reduce premature CVD mortality using a three-pronged approach: improve blood pressure control rates, reduce population salt intake, and eliminate artificial trans-fat in the diet. In March 2020, due to restrictions imposed by the COVID-19 pandemic, the program was scaled back from 12 regions to a demonstration project in a single district, District 1 of Iloilo Province, in a single region, the Western Visayas (Region VI). The project was implemented through seven Rural Health Units (RHUs) in each of the seven municipalities of District 1. An RHU is an outpatient care facility that offers routine primary healthcare (PHC) services to rural and underserved communities. It is staffed by formal cadres of health workers—physicians, nurses, and midwives— as well as barangay health workers who act as the first point of contact between the healthcare system and the community. Local government units (LGUs) in the Philippines are given the autonomy and responsibility for managing and implementing their health programs and services. The Department of Health (DOH) provides technical support and guidelines. In this arrangement, provincial governments manage and operate primary and secondary-level hospital services through district and provincial hospitals. Municipal governments provide primary care, including preventive and promotive health services, and other public health programs through rural health units (RHUs), health centers, and barangay health stations (BHSs) [4].

The Healthy Hearts demonstration project articulates components of a scalable provincial model for the delivery of HTN services at the PHC level which are aligned with the recommendations outlined in: a) the HEARTS Technical Package which promotes adherence to standardized simple treatment protocols and lifestyle counseling [5] the Philippines Universal Health Care (UHC) Act which came into effect in 2020 [6]. The latter aims to integrate fragmented local health systems into province- and city-wide health systems and further decentralize health system financing to local government units (LGUs). Of particular significance, the central Department of Health (DOH) phased out the procurement and financing of antihypertensive medicines in 2022, placing the full responsibility for supplying these medicines on LGUs, at no out-of-pocket cost to patients [7].

Under UHC, RHUs must be accredited by PhilHealth, the national health insurance corporation, to qualify for capitation funds [8]. Among other requirements, RHUs must have the capability to offer FDA-licensed pharmacy services, either in-house or outsourced. Since many RHUs do not have pharmacists or pharmacy assistants, and may have insufficient space for storage and dispensing of medicines, a model was piloted to outsource pharmacy services to private pharmacies located in the catchment area of two of the seven RHUs participating in the Healthy Hearts demonstration project. The prices for the three hypertension medicines specified in the national treatment protocol, generic amlodipine, losartan, and hydrochlorothiazide, were negotiated between the LGUs and the pharmacies and were inclusive of all logistics costs plus a dispensing fee. This outsourcing model aimed to explore potential operational efficiencies in RHUs where in-house pharmacy services are currently not feasible. In this model, patients would not need to go to the RHU to obtain their hypertension medication, which would eliminate need for storage and handling by healthcare providers; instead, patients would obtain medications from unaffiliated pharmacies.

In this paper, we estimated the total and per-patient costs of implementing the Healthy Hearts provincial model for the delivery of HTN services in 7 RHUs in Iloilo province, distinguishing between provider costs and medication costs under three different medicine procurement scenarios: local government procurement, national pooled procurement, and outsourced procurement through private pharmacies. Using data from the participating RHUs, annual total costs were projected to the district and province levels. Understanding the key cost drivers associated with individual components of the Healthy Hearts service provision model can help policymakers optimize the delivery of HTN services, assess the scalability of the model under different medicine procurement schemes, and plan annual budgets in the context of the 2019 UHC Act.

## Methods

### Setting

**Fig. 1:**
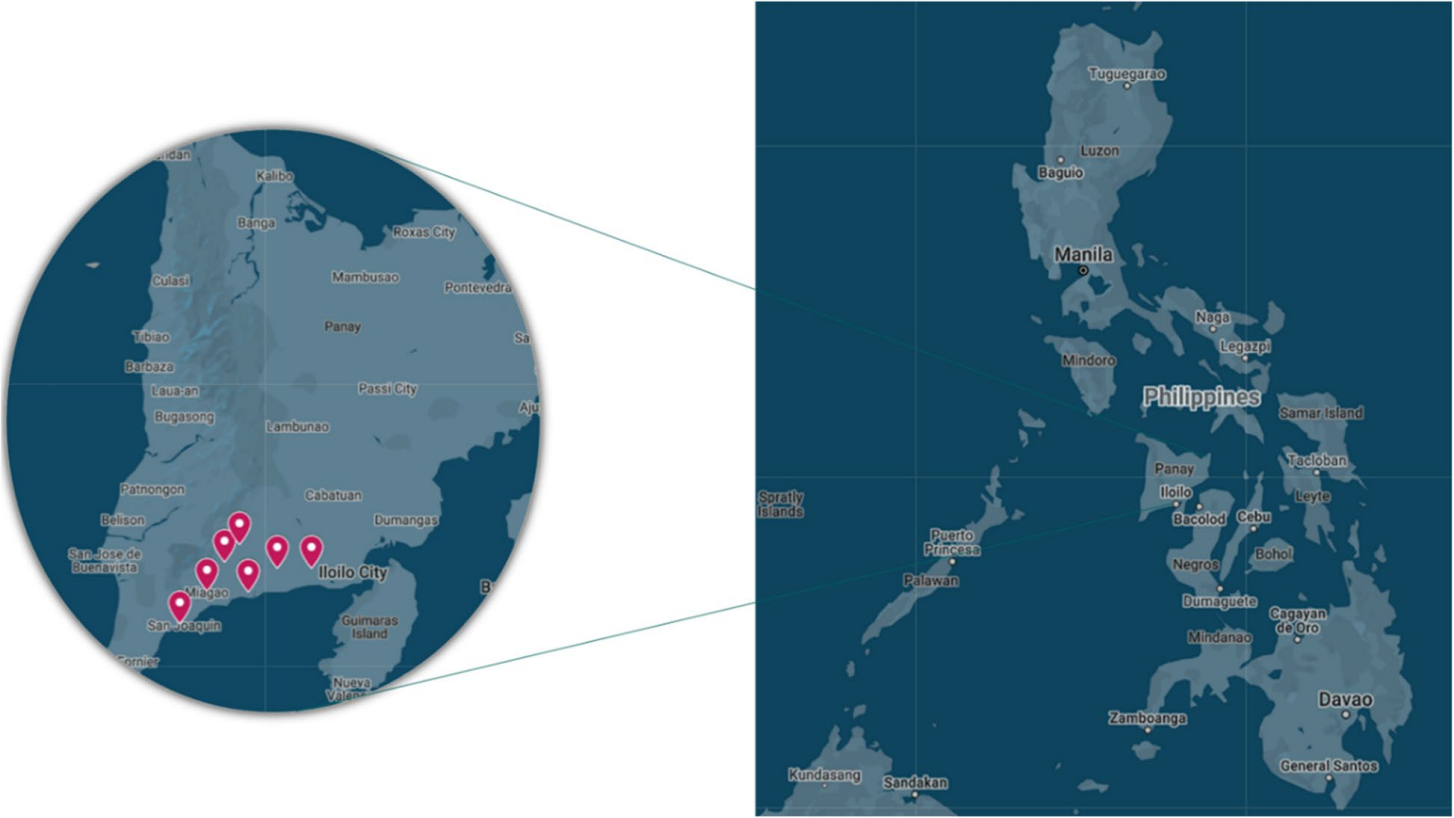
Municipalities in District 1 of Iloilo Province as RHU study sites [9]

This study was conducted at the seven RHUs of the seven municipalities of District 1 of Iloilo Province in the Western Visayas region of the Philippines: Guimbal, Igbaras, Oton, Miag-ao, San Joaquin, Tigbauan, and Tubungan (Fig. 1). District 1 of Iloilo Province is characterized by a predominantly agricultural economy with a relatively young population with good access to education. Healthcare services are accessible through Rural Health Units (RHUs) and various health programs, such as the HEARTS hypertension program, which aim to improve public health outcomes.

**Fig. 2:**
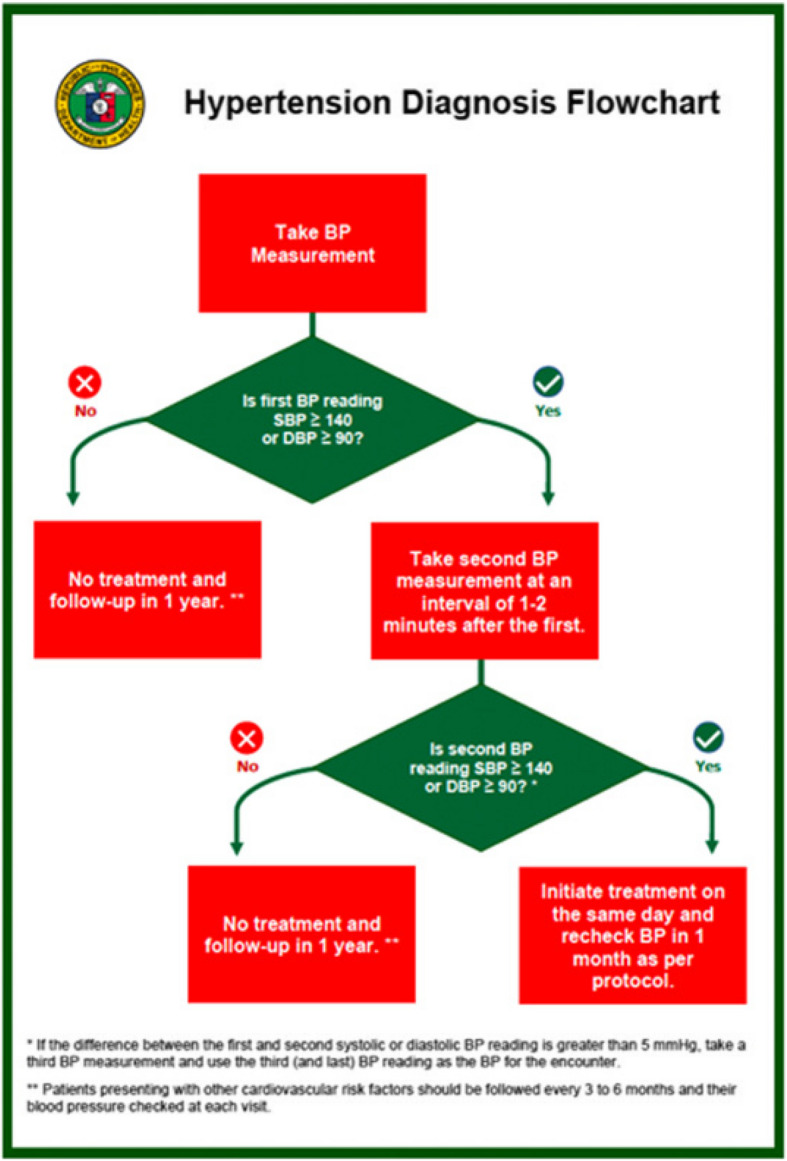
Hypertension Diagnosis Flowchart

**Fig. 3:**
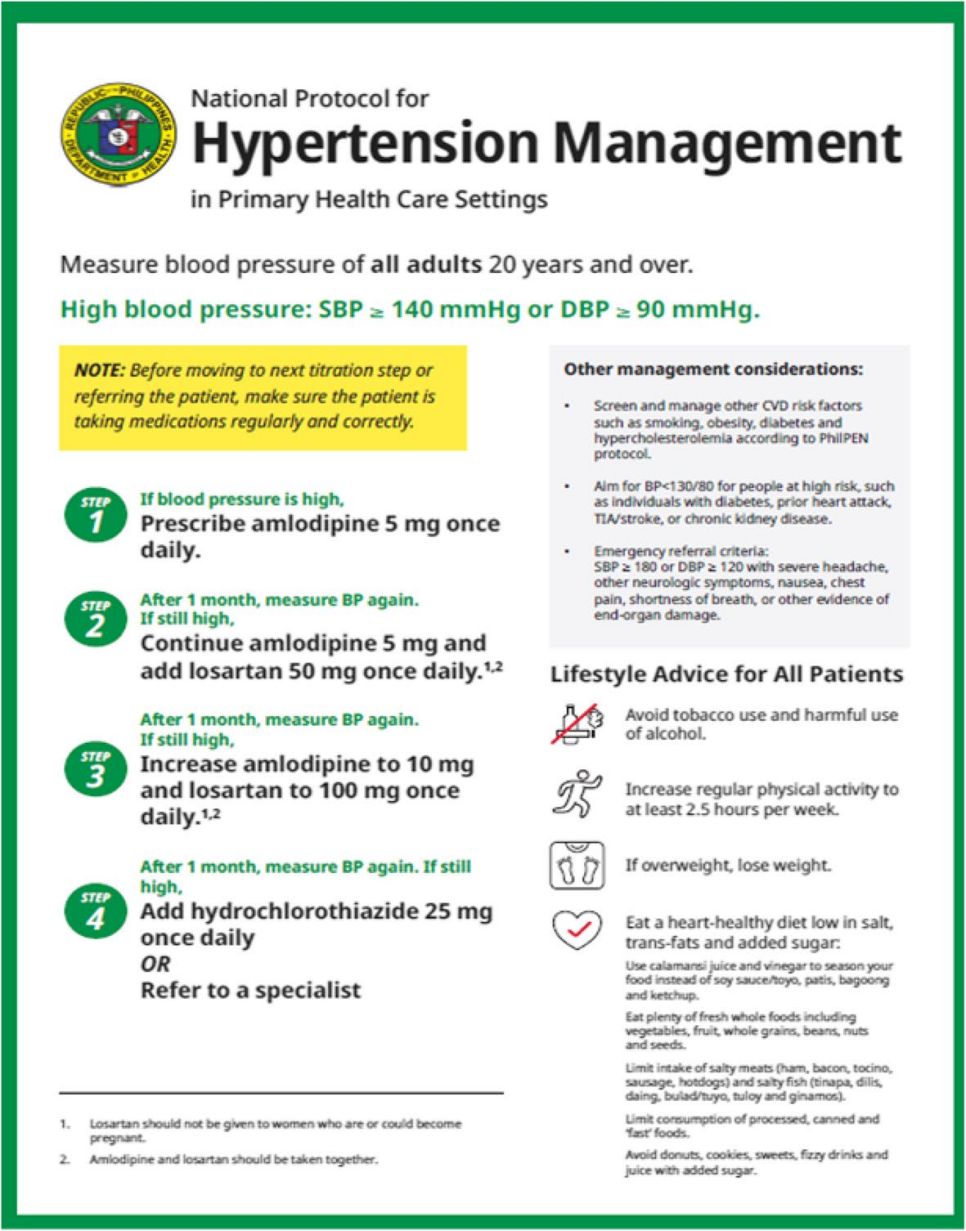
Philippine National Protocol for Hypertension Management in Primary Health Care Settings

All items in the Philippines National Protocol for Hypertension Management were applied in the Healthy Hearts demonstration project, including: a) the Blood Pressure Measurement Checklist; b) the Hypertension Diagnosis Flowchart (Fig. 2); c) the National Protocol for Hypertension Management in Primary Health Care Settings (Fig. 3); d) the facility electronic HTN registry (e-Registry); and e) a self-paced modular eLearning course on the Prevention and Management of Hypertension housed on the DOH Academy platform. All patients presenting at the RHUs are screened for hypertension by the nurse or nurse midwife, and those found to have a blood pressure (BP) equal to or higher than 140/90 mmHg are referred to the Medical Health officer for hypertension diagnosis and management, and enrolled in the e-Registry.

The national treatment protocol consists of four steps (Fig. 3). In Step 1, The first line of antihypertensive treatment is amlodipine 5 mg once a day. If the BP is uncontrolled after a month, the second line of treatment (Step 2) is to continue taking amlodipine 5 mg and adding losartan 50 mg once daily. If the BP is still uncontrolled after a month, the third-line antihypertensive treatment (Step 3) entails increasing amlodipine to 10 mg and losartan to 100 mg once daily. After 1 month, if the BP is still uncontrolled, hydrochlorothiazide 25 mg once a day is added or the patient is referred to a specialist (Step 4) [1, 10].

### Cost estimation

The HEARTS costing tool is a Microsoft Excel-based instrument for gathering, analyzing, and evaluating data on the incremental cost of implementing primary care hypertension programs from a health system's perspective [11]. The major cost components for the Healthy Hearts program, a program for hypertension management in the Philippines' primary care and universal care contexts, are the costs of provider time and the costs of treatment (i.e., the cost of medications). Other program cost components include costs related to training, miscellaneous logistics, and systems for monitoring. However, the objectives of this study entailed a comparative assessment of some specific scenarios about medicine and provider time costs, and the study focuses on these aspects instead of assessing all the program cost components. To align with our study's objectives and the context of the Healthy Hearts Demonstration Project in the Philippines, we restructured the HEARTS costing tool, streamlining the evaluation components and estimating total costs and costs per patient for two main categories – medication costs and provider time costs.

#### Provider costs

Provider costs reflect provider time used in following the Philippines National Hypertension Treatment Protocol. These costs were calculated based on total provider time (in minutes) for performing each task under the protocol, the average salary including benefits of the provider performing the task (weighted salary for the nurse/nurse-midwife category), and the number of follow-up visits required every year.

Provider tasks included BP measurement, medical consultation, lifestyle counseling, dispensing or refilling medications, enrolling a new patient, and adding a visit in the e-Registry. Provider tasks vary depending on whether a patient visit is a first (enrollment) visit or a follow-up visit (Table 1). Notably, provider time for dispensing or refilling medications is zero when medicines are outsourced to private pharmacies. In this model, HTN services depend on BP control status. Patients with uncontrolled BP require 12 monthly visits per annum while those with controlled BP only require 4 quarterly visits per annum.

**Table 1:** Input costs, cost parameters, and cost assumptions

Input description	Units	Guimbal	Igbaras	Miagao	Oton	San Joaquin	Tigbauan	Tubungan
Total population > 20 years	Persons	21,714	19,962	42,231	61,076	32,623	40,452	14,273
Patients with hypertension (e-Registry)	Persons	3,006	1,205	2,729	3,226	4,757	4,014	1,383
**Patient distribution**								
** Treatment protocol step 1**								
Patients with hypertension	Percent	51%	68%	76%	70%	62%	44%	44%
Patients with HTN under control	Percent	31%	41%	46%	42%	37%	27%	26%
Patients with HTN not under control	Percent	20%	27%	31%	28%	25%	18%	18%
** Treatment protocol step 2**								
Patients with hypertension	Percent	46%	25%	19%	27%	34%	44%	47%
Patients with HTN under control	Percent	16%	9%	6%	10%	12%	15%	16%
Patients with HTN not under control	Percent	30%	16%	12%	18%	22%	29%	30%
** Treatment protocol step 3**								
Patients with hypertension	Percent	3%	7%	5%	2%	4%	12%	9%
Patients with HTN under control	Percent	0%	0%	0%	0%	0%	1%	0%
Patients with HTN not under control	Percent	3%	6%	5%	2%	4%	11%	9%
**Number of visits**								
Patients with HTN under control	visits/year	4	4	4	4	4	4	4
Patients with HTN not under control	visits/year	12	12	12	12	12	12	12
**Medicine price**								
** Amlodipine 5 mg**								
Baseline	PHP/tablet	0.65	0.65	0.65	0.65	0.65	0.65	0.65
Scenario 1	PHP/tablet	0.194	0.194	0.194	0.194	0.194	0.194	0.194
Scenario 2	PHP/tablet	1.975	1.975	1.975	1.95	1.975	2	1.975
** Losartan 50 mg**								
Baseline	PHP/tablet	1.67	1.67	1.67	1.67	1.67	1.67	1.67
Scenario 1	PHP/tablet	0.562	0.562	0.562	0.562	0.562	0.562	0.562
Scenario 2	PHP/tablet	3.375	3.375	3.375	2.75	3.375	4	3.375
** Amlodipine 10 mg**								
Baseline	PHP/tablet	1.3	1.3	1.3	1.3	1.3	1.3	1.3
Scenario 1	PHP/tablet	0.388	0.388	0.388	0.388	0.388	0.388	0.388
Scenario 2	PHP/tablet	3.95	3.95	3.95	3.9	3.95	4	3.95
** Losartan 100 mg**								
Baseline	PHP/tablet	3.34	3.34	3.34	3.34	3.34	3.34	3.34
Scenario 1	PHP/tablet	1.124	1.124	1.124	1.124	1.124	1.124	1.124
Scenario 2	PHP/tablet	6.75	6.75	6.75	5.5	6.75	8	6.75
**Wages/Salaries**								
Doctor	PHP/year	850,237	840,000	1,116,000	1,044,000	1,104,000	951,906	840,000
Nurses	PHP/year	520,430	543,830	636,300	537,830	528,000	532,944	520,840
Midwife	PHP/year	321,673	430,000	313,300	424,000	256,000	378,680	326,344
BHW	PHP/year	13,600	17,332	10,600	41,200	24,600	17,700	20,800
Pharmacist	PHP/year	255,394	186,820	186,820	186,820	186,820	186,820	329,789
Encoder	PHP/year	108,000	108,000	117,600	113,000	94,800	100,800	117,600
**Provider time per patient by activity**								
** First-visit**								
BP measurement	Minutes/patient/visit	5	5	5	5	5	5	5
Medical Consultation (Hx & PE)	Minutes/patient/visit	15	15	15	15	15	15	15
Counseling	Minutes/patient/visit	10	10	10	10	10	10	10
Dispensing medications^a^	Minutes/patient/visit	3	3	3	3	3	3	3
Enrolling new patients	Minutes/patient/visit	1	1	1	1	1	1	1
Recording visit	Minutes/patient/visit	1	1	1	1	1	1	1
** Follow-up visit**								
BP measurement	Minutes/patient/visit	5	5	5	5	5	5	5
Medical Consultation^b^	Minutes/patient/visit	15	15	15	15	15	15	15
Counseling	Minutes/patient/visit	10	10	10	10	10	10	10
Medication refill^a^	Minutes/patient/visit	5	5	5	5	5	5	5
Recording visit	Minutes/patient/visit	1	1	1	1	1	1	1
**Task Sharing by provider by activity (First visit)**								
** BP measurement**								
Nurses	Percent	50%	45%	45%	30%	30%	25%	25%
Midwife	Percent	50%	45%	45%	30%	30%	25%	25%
BHW	Percent		10%	10%	40%	40%	50%	50%
** Medical Consultation (Hx & PE)**								
Doctor	Percent	100%	100%	100%	100%	100%	100%	100%
** Counseling**								
Nurses	Percent	50%	40%	35%	30%	25%	25%	40%
Midwife	Percent	50%	40%	35%	30%	25%	25%	40%
BHW	Percent		20%	30%	40%	30%	50%	20%
** Dispensing medications**^**a**^								
Nurses	Percent	15%	40%	5%	50%	50%	50%	
Midwife	Percent	15%	40%	5%	50%	50%	50%	
BHW	Percent		20%	10%				
Pharmacist	Percent	70%		80%				100%
** Enrolling new patients**								
Nurses	Percent	30%	5%	25%	45%			
Midwife	Percent	30%	5%	25%	45%			
Encoder	Percent	40%	90%	50%	10%	100%	100%	100%
** Recording visit**								
Nurses	Percent		5%	25%	10%		0%	0%
Midwife	Percent		5%	25%	10%		0%	0%
BHW	Percent					20%		
Encoder	Percent	100%	90%	50%	80%	80%	100%	100%
**Task Sharing by provider by activity (Follow-up visit)**								
** BP measurement**								
Nurses	Percent	50%	45%	45%	20%		25%	50%
Midwife	Percent	50%	45%	45%	20%		25%	50%
BHW	Percent		10%	10%	60%	100%	50%	
** Medical Consultation**^**b**^								
Doctor	Percent	100%	100%	100%	100%	100%	100%	100%
** Counseling**								
Nurses	Percent	50%	40%	35%	20%		25%	40%
Midwife	Percent	50%	40%	35%	20%		25%	40%
BHW	Percent		20%	30%	60%	100%	50%	20%
** Dispensing medications**^**a**^								
Nurses	Percent	15%	40%	5%	20%		50%	
Midwife	Percent	15%	40%	5%	20%		50%	
BHW	Percent		20%	10%	60%	100%		
Pharmacist	Percent	70%		80%				100%
** Recording visit**								
Nurses	Percent		5%	25%	10%			
Midwife	Percent		5%	25%	10%			
BHW	Percent					20%		
Encoder	Percent	100%	90%	50%	80%	80%	100%	100%

^a^This activity is absent in Scenario 2 (outsourced medication procurement)

^b^Applies only to patients with uncontrolled HTN at follow-up visits

#### Medication costs

The cost of medicines is restricted to those stipulated in the Philippine National Protocol for Hypertension Management in Primary Health Care Settings: amlodipine, losartan, and hydrochlorothiazide [10]. Since most patients fall within steps 1–3 of the national protocol, which exclude hydrochlorothiazide, the price of hydrochlorothiazide was not included in the study. Medications for treating diabetes and dyslipidemia or other co-morbidities such as atrial fibrillation, congestive heart failure, angina or myocardial infarction, were also excluded. Three medicine procurement scenarios were costed:

- Baseline Scenario: Local procurement in which individual LGUs procure the medicines.
- Scenario 1: Pooled procurement in which multiple LGUs pool their resources with the Philippine Pharma Procurement Inc. (PPPI) national pooling mechanism, a national purchasing entity [12].
- Scenario 2: Outsourced procurement in which LGUs outsource pharmacy services to private pharmacies in their catchment area.

The population used for estimating cost included patients aged 20 years and older, registered in one of the seven HTN e-Registries, and actively receiving hypertension care in the demonstration project.

Annual per-patient costs at the district level were calculated as the weighted average of per-patient costs across the seven RHUs. After obtaining the per-patient annual cost of delivering HTN services at each RHU, total costs were projected to the district and province levels using their respective patient populations. To project the total annual public sector costs to the province level, district per-patient costs were multiplied by the estimated number of patients in the province. The estimated number of patients in the province was imputed by multiplying the proportion of registered patients in District 1 (8.75% of the district’s population 20 years and older) by the number of persons 20 years and older in Iloilo province.

### Sources of data

Data on the population and number of patients with HTN (controlled and uncontrolled) were obtained from the DOH e-Registry for each municipality. Data on time required to perform each task and data on salaries were obtained through key informant interviews (KIIs) and focus group discussions (FGDs). KIIs were conducted with each of the seven Municipal Health Officers (MHO). FGDs were conducted at each site among a total of three to five nurses, midwives, pharmacists, laboratory technicians, or community health workers. The objective of the KIIs and FGDs was not a qualitative assessment of program implementation, and therefore qualitative information was not collected or recorded. Only quantitative cost inputs were recorded and subsequently entered into the Excel costing tool file, as described in Table 1.

Medicine prices for the ‘baseline’ procurement scenario were obtained from a Request For Quotation (RFQ) submitted in 2021 for amlodipine and losartan. Prices for the ‘pooled’ procurement were obtained from the Philippine Pharma Procurement Inc. (PPPI) plus 8% to include logistics costs. Prices for the ‘outsourced’ scenario were the prices negotiated respectively by the LGUs of Oton and Tigbauan and private pharmacies in their catchment areas. In this scenario, RHUs outside the Oton and Tigbauan municipalities were assigned the average outsourced prices of the Oton and Tigbauan RHUs.

### Cost model inputs

A summary of the model inputs is presented in Table 1. At the time of the study, there were 20,320 hypertensive patients enrolled in the Healthy Hearts demonstration project. Their distributions across the seven RHUs and across the four steps of the national treatment protocol according to their HTN status (controlled or uncontrolled) are provided in Table 1. Nearly all patients received treatment with regimens from protocol steps 1–3, with control rates ranging from zero to 46% depending on the regimen group and location. As per the Philippines National HTN treatment protocol, controlled HTN was defined as a SBP < 140 mmHg and DBP < 90, and uncontrolled HTN was defined as SBP ≥ 140 mmHg or DBP ≥ 90 mmHg among patients on treatment.

The baseline prices for amlodipine 5 mg and 10 mg, losartan 50 mg and 100 mg were collected from each RHU during field visits, while prices for Scenario 1 (pooled procurement) were gathered from the Iloilo Provincial Health Office. Medicine prices for Scenario 2 (outsourced procurement) were collected from the outsourcing pharmacies in Oton and Tigbauan, and the average values of these prices were applied for the rest of the RHUs. Data on wages/salaries of the health professionals and workers, provider time per patient by activity, and the task sharing by the provider by activity were collected during the KII and FGD. Costs were reported in US Dollars (USD) and Philippine pesos (PhP), using a currency conversion rate of PhP 58.87 for 1 USD [13].

## Results

### Medicine costs

The annual total and per-patient medicines costs for seven RHUs are summarized in Table 2. Medicine costs were lower across the seven RHUs when procurement was pooled (scenario 1) compared to local procurement (baseline). Medicine costs were considerably higher than baseline when procurement was outsourced to private pharmacies (scenario 2).

**Table 2:** Annual total and per-patient medicine costs across 7 RHUs, Iloilo Province District 1, under different medicine procurement scenarios

		***Total medicine cost***		***Medicine cost per patient***	
		PHP	USD	PHP	USD
Guimbal	Baseline	1,687,369	28,663	561	9.5
	Scenario 1	539,883	9,171	180	3.1
	Scenario 2	4,157,527	70,622	1,383	23.5
	Difference: Scenario 1—Baseline	−1,147,485	−19,492	−382	−6.5
	Difference: Scenario 2—Baseline	2,470,159	41,960	822	14.0
Igbaras	Baseline	589,272	10,010	489	8.3
	Scenario 1	186,692	3,171	155	2.6
	Scenario 2	1,501,335	25,503	1,246	21.2
	Difference: Scenario 1—Baseline	−402,580	−6,838	−334	−5.7
	Difference: Scenario 2—Baseline	912,064	15,493	757	12.9
Miagao	Baseline	1,155,013	19,620	423	7.2
	Scenario 1	362,814	6,163	133	2.3
	Scenario 2	3,025,974	51,401	1,109	18.8
	Difference: Scenario 1—Baseline	−792,198	−13,457	−290	−4.9
	Difference: Scenario 2—Baseline	1,870,961	31,781	686	11.6
Oton	Baseline	1,416,455	24,061	439	7.5
	Scenario 1	446,814	7,590	139	2.4
	Scenario 2	3,394,116	57,654	1,052	17.9
	Difference: Scenario 1—Baseline	−969,641	−16,471	−301	−5.1
	Difference: Scenario 2—Baseline	1,977,662	33,594	613	10.4
San Joaquin	Baseline	2,384,412	40,503	501	8.5
	Scenario 1	757,764	12,872	159	2.7
	Scenario 2	6,012,454	102,131	1,264	21.5
	Difference: Scenario 1—Baseline	−1,626,649	−27,631	−342	−5.8
	Difference: Scenario 2—Baseline	3,628,042	61,628	763	13.0
Tigbauan	Baseline	2,710,770	46,047	675	11.5
	Scenario 1	871,791	14,809	217	3.7
	Scenario 2	7,217,388	122,599	1,798	30.5
	Difference: Scenario 1—Baseline	−1,838,979	−31,238	−458	−7.8
	Difference: Scenario 2—Baseline	4,506,618	76,552	1123	19.1
Tubungan	Baseline	904,618	15,366	654	11.1
	Scenario 1	290,816	4,940	210	3.6
	Scenario 2	2,192,065	37,236	1,585	26.9
	Difference: Scenario 1—Baseline	−613,802	−10,426	−444	−7.5
	Difference: Scenario 2—Baseline	1,287,447	21,869	931	15.8

Baseline scenario: local government procurement; Scenario 1: national pooled procurement; Scenario 2: outsourced private pharmacy procurement

The per-patient medicine costs under local procurement ranged from PhP 423 (USD 7.2) to PhP 675 (USD 11.5) across the 7 RHUs. The weighted average across the RHUs in the local procurement scenario was Php 534 (USD 9.1), which was higher than the national procurement scenario Php 170 (USD 2.9) and lower than the outsourced pharmacy scenario Php 1353 (USD 23).

### Provider costs

The annual per-patient and total provider costs for the seven RHUs are presented in Tables 3 and 4 respectively, by HTN control status. Provider costs across the seven RHUs were consistently higher among patients with uncontrolled HTN (who require monthly follow-up visits with medical consultation) compared to those with controlled HTN (who require quarterly follow-up visits without medical consultation).

**Table 3:** Annual per-patient provider costs across 7 RHUs, Iloilo Province District 1, by HTN control status

		**Per patient annual provider cost**					
		**All patients**		**Controlled**		**Uncontrolled**	
		PHP	USD	PHP	USD	PHP	USD
Guimbal	Baseline	1,214	20.6	346	5.9	1,980	33.6
	Scenario 1	1,214	20.6	346	5.9	1,980	33.6
	Scenario 2	1,118	19.0	301	5.1	1,837	31.2
	Difference: Scenario 1—Baseline	0	0	0	0.0	0	0
	Difference: Scenario 2—Baseline	−97	−2	−45	−0.8	−143	−2
Igbaras	Baseline	1,180	20.0	360	6.1	1,999	34.0
	Scenario 1	1,180	20.0	360	6.1	1,999	34.0
	Scenario 2	1,060	18.0	301	5.2	1,816	30.8
	Difference: Scenario 1—Baseline	0	0	0	0.0	0	0
	Difference: Scenario 2—Baseline	−120	−2	−59	−1.0	−183	−3
Miagao	Baseline	1,256	21.3	351	6.0	2,261	38.4
	Scenario 1	1,256	21.3	351	6.0	2,261	38.4
	Scenario 2	1,197	20.3	322	5.5	2,169	36.8
	Difference: Scenario 1—Baseline	0	0	0	0.0	0	0
	Difference: Scenario 2—Baseline	−59	−1	−29	−0.5	−92	−2
Oton	Baseline	1,092	18.6	288	4.9	1,958	33.3
	Scenario 1	1,092	18.6	288	4.9	1,958	33.3
	Scenario 2	1,021	17.3	250	4.2	1,851	31.4
	Difference: Scenario 1—Baseline	0	0	0	0.0	0	0
	Difference: Scenario 2—Baseline	−71	−1	−38	−0.6	−107	−1.8
San Joaquin	Baseline	959	16.3	201	3.4	1,697	28.8
	Scenario 1	959	16.3	201	3.4	1,697	28.8
	Scenario 2	942	16.0	189	3.2	1,677	28.5
	Difference: Scenario 1—Baseline	0	0	0	0.0	0	0.0
	Difference: Scenario 2—Baseline	−16	−0.28	−12	−0.21	−20	−0.34
Tigbauan	Baseline	1,239	21.0	298	5.1	1,937	32.9
	Scenario 1	1,239	21.0	298	5.1	1,937	32.9
	Scenario 2	1,089	18.5	232	3.9	1,725	29.3
	Difference: Scenario 1—Baseline	0	0	0	0	0	0
	Difference: Scenario 2—Baseline	−150	−3	−66	−1	−212	−4
Tubungan	Baseline	1,219	20.7	324	5.5	1,902	32.3
	Scenario 1	1,219	20.7	324	5.5	1,902	32.3
	Scenario 2	1,111	18.9	277	4.7	1,749	29.7
	Difference: Scenario 1—Baseline	0	0	0	0.0	0	0.0
	Difference: Scenario 2—Baseline	−107	−2	−48	−1	−153	−3

**Table 4:** Annual total provider costs across 7 RHUs, Iloilo Province District 1, by HTN control status

		**Total annual provider cost**					
		**All patients**		**Controlled**		**Uncontrolled**	
		**PHP**	**USD**	**PHP**	**USD**	**PHP**	**USD**
**Guimbal**	**Baseline**	**3,650,543**	**62,010**	**487,443**	**8,280**	**3,163,099**	**53,730**
	**Scenario 1**	**3,650,543**	**62,010**	**487,443**	**8,280**	**3,163,099**	**53,730**
	**Scenario 2**	3,359,546	57,067	424,306	7,208	2,935,240	49,860
	Difference: Scenario 1—Baseline	0	0	0	0.0	0	0
	Difference: Scenario 2—Baseline	−290,996	−4,943	−63,138	−1,072	−227,859	−3,871
**Igbaras**	**Baseline**	**1,421,300**	**24,143**	**216,838**	**3,683**	**1,204,462**	**20,460**
	**Scenario 1**	**1,421,300**	**24,143**	**216,838**	**3,683**	**1,204,462**	**20,460**
	**Scenario 2**	1,277,094	21,693	182,692	3,103	1,094,401	18,590
	Difference: Scenario 1—Baseline	0	0	0	0.0	0	0
	Difference: Scenario 2—Baseline	−144,206	−2,450	−34,146	−580	−110,061	−1,870
**Miagao**	**Baseline**	**3,427,846**	**58,227**	**503,574**	**8,554**	**2,924,272**	**49,673**
	**Scenario 1**	**3,427,846**	**58,227**	**503,574**	**8,554**	**2,924,272**	**49,673**
	**Scenario 2**	3,267,840	55,509	462,575	7,858	2,805,265	47,652
	Difference: Scenario 1—Baseline	0	0	0	0	0	0
	Difference: Scenario 2—Baseline	−160,006	−2,718	−40,999	−696	−119,007	−2,022
**Oton**	**Baseline**	**3,523,262**	**59,848**	**480,993**	**8,170**	**3,042,269**	**51,678**
	**Scenario 1**	**3,523,262**	**59,848**	**480,993**	**8,170**	**3,042,269**	**51,678**
	**Scenario 2**	**3,293,696**	**55,949**	**418,024**	**7,101**	**2,875,672**	**48,848**
	Difference: Scenario 1—Baseline	0	0	0	0	0	0
	Difference: Scenario 2—Baseline	−229,566	−3,900	−62,969	−1,070	−166,597	−2,830
**San Joaquin**	**Baseline**	**4,561,247**	**77,480**	**472,179**	**8,021**	**4,089,068**	**69,459**
	**Scenario 1**	**4,561,247**	**77,480**	**472,179**	**8,021**	**4,089,068**	**69,459**
	**Scenario 2**	4,483,362	76,157	443,111	7,527	4,040,252	68,630
	Difference: Scenario 1—Baseline	0	0	0	0.0	0	0
	Difference: Scenario 2—Baseline	−77,885	−1,323	−29,068	−494	−48,817	−829
**Tigbauan**	**Baseline**	**4,973,163**	**84,477**	**509,378**	**8,653**	**4,463,785**	**75,824**
	**Scenario 1**	**4,973,163**	**84,477**	**509,378**	**8,653**	**4,463,785**	**75,824**
	**Scenario 2**	**4,372,560**	**74,275**	**397,010**	**6,744**	**3,975,551**	**67,531**
	Difference: Scenario 1—Baseline	0	0	0	0	0	0
	Difference: Scenario 2—Baseline	−600,602	−10,202	−112,368	−1,909	−488,234	−8,293
**Tubungan**	**Baseline**	**1,685,499**	**28,631**	**194,404**	**3,302**	**1,491,094**	**25,329**
	**Scenario 1**	**1,685,499**	**28,631**	**194,404**	**3,302**	**1,491,094**	**25,329**
	**Scenario 2**	1,536,870	26,106	165,901	2,818	1,370,969	23,288
	Difference: Scenario 1—Baseline	0	0	0	0	0	0
	Difference: Scenario 2—Baseline	−148,628	−2,525	−28,503	−484	−120,125	−2041

In all RHUs, there was no difference in provider costs between the local procurement (baseline) and pooled procurement (Scenario 1) since provider activity in these two scenarios is not affected by whether medications are procured locally or at pooled prices. However, in the outsourced pharmacy scenario (Scenario 2), annual provider costs were lower than baseline, reflecting savings from the absence of dispensing time and costs when medicines are outsourced.

Under the local and pooled procurement scenarios, the per-patient provider costs ranged from PhP 201 (USD 3.4) and Php360 (USD6.1) for controlled patients, and PhP 1697 (USD 28.8) and PhP 2261 (USD 38.4) for uncontrolled patients across the seven RHUs. Under the outsourced pharmacy scenario, the per-patient provider costs ranged from PhP 189 (USD 3.2) to PhP 322 (USD5.5) for controlled patients and from PhP 1677 (USD 28.5) and PhP 2169 (USD 36.8) for uncontrolled patients across the seven RHUs.

### Medicine costs projected to the district and province levels

The annual per-patient and total medicine costs projected to the district and province levels are presented in Table 5. Per-patient medicine costs, calculated as a weighted average across the 7 district RHUs, were estimated to be lowest under the pooled procurement scenario at PhP 170 (USD 2.9) per patient, highest under the outsourced pharmacy scenario at Php 1353 (USD 23) per patient, and in between under the local procurement scenario at Php 534 (USD 9.1) per patient.

**Table 5:** Annual per-patient and total medicine costs projected to the district and province levels

	**Medicine Cost**			
	**Medicine cost per patient**		**Total medicine cost**	
	**PHP**	**USD**	**PHP**	**USD**
	**District Level**			
**Baseline**	534	9.1	10,847,908	184,269
**Scenario 1**	170	2.9	3,456,574	58,715
**Scenario 2**	1353	23.0	27,500,860	467,146
Difference: Scenario 1—Baseline	−364	−6.2	−7,391,334	−125,553
Difference: Scenario 2—Baseline	820	13.9	16,652,952	282,877
	**Province Level**			
**Baseline**	534	9.1	59,400,230	1,009,007
**Scenario 1**	170	2.9	18,927,270	321,510
**Scenario 2**	1353	23.0	150,587,327	2,557,964
Difference: Scenario 1—Baseline	−364	−6.2	−40,472,960	−687,497
Difference: Scenario 2—Baseline	820	13.9	91,187,097	1,548,957

At the district level, the total medicine cost of PhP 3.46 million (USD 58,715) in Scenario 1 was lower compared to the baseline of PhP 10.8 million (USD 184,269). Under Scenario 2, the total medicine cost of PhP 27.5 million (USD 467,146) was higher than the baseline. At the province level, the total medicine cost of PhP 18.9 million (USD 321,510) in Scenario 1 was also lower compared to the baseline. Under Scenario 2, the total medicine cost of PhP 150.6 million (USD 2.6 million) was higher compared to the baseline (Table 5).

### Provider costs projected to the district and province levels

The district-level provider cost per patient, estimated as the weighted average of the per-patient provider costs of the district’s 7 RHUs, is listed in Table 6. This per-patient cost was used to project annual total provider costs at the district and province levels, which are presented in Table 7. Both per-patient and total provider costs are dramatically higher in patients with uncontrolled HTN because of the threefold higher number of follow-up visits required compared to patients with controlled HTN. For example, the baseline per-patient annual provider cost was PhP 1,144 (USD 19.4) across all patients, but it was only PhP 293 (USD 5) for patients with controlled HTN and PhP 1,935 (USD 33) for patients with uncontrolled HTN.

**Table 6:** Annual per-patient provider costs by HTN control status, district-level weighted average across 7 RHUs

	**Per patient annual provider cost**					
	**All patients**		**Controlled**		**Uncontrolled**	
	**PHP**	**USD**	**PHP**	**USD**	**PHP**	**USD**
**Baseline**	1,144	19.4	293	5.0	1,935	32.9
**Scenario 1**	1,144	19.4	293	5.0	1,935	32.9
**Scenario 2**	1,063	18.0	254	4.3	1,817	30.9
Difference: Scenario 1—Baseline	0	0.0	0	0.0	0	0.0
Difference: Scenario 2—Baseline	−81	−1.4	−39	−0.7	−118	−2.0

**Table 7:** Annual total provider costs by HTN control status, projected to the district and province levels

	**Total annual provider cost**					
	**All patients**		**Controlled**		**Uncontrolled**	
	PHP	USD	PHP	USD	PHP	USD
**District Level**						
** Baseline**	23,242,858	394,817	2,864,808	48,663	20,378,050	346,153
** Scenario 1**	23,242,858	394,817	2,864,808	48,663	20,378,050	346,153
** Scenario 2**	21,590,968	366,757	2,493,618	42,358	19,097,349	324,399
Difference: Scenario 1—Baseline	0	0	0	0	0	0
Difference: Scenario 2—Baseline	−1,651,891	−28,060	−371,190	−6,305	−1,280,701	−21,755
**Province Level**						
** Baseline**	127,271,654	2,161,910	15,686,922	266,467	111,584,732	1,895,443
** Scenario 1**	127,271,654	2,161,910	15,686,922	266,467	111,584,732	1,895,443
** Scenario 2**	118,226,343	2,008,261	13,654,383	231,941	104,571,960	1,776,320
Difference: Scenario 1—Baseline	0	0	0	0	0	0
Difference: Scenario 2—Baseline	−9,045,311	−153,649	−2,032,539	−34,526	−7,012,772	−119,123

As observed previously, provider costs decrease modestly across the board when medicines are outsourced to private pharmacies, reflecting the savings from eliminating in-house medication dispensing and refill activities. Per-patient provider costs decrease slightly from Php 1,144 (USD 19.40) to Php 1,063 (USD 18.0) per patient when medicine procurement is outsourced (Table 6).

### Medicine costs, by medication

**Fig. 4:**
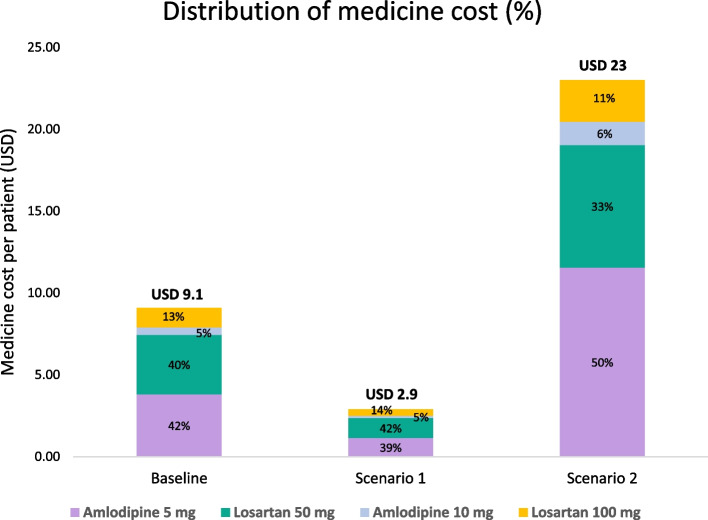
Distribution of medicine cost by medication

Figure 4 explores the relative contribution of different medications to per-patient medicine costs. This distribution does not vary widely by scenario. Amlodipine 5 mg represents the highest share of medicine cost, owing in part to amlodipine being the step 1 medication prescribed in the Philippines HTN treatment protocol, followed closely by the step 2 medication, losartan 50 mg. Amlodipine 10 mg, prescribed at protocol step 3, had the lowest percentage of total medication costs.

### Provider costs, by activity

**Fig. 5:**
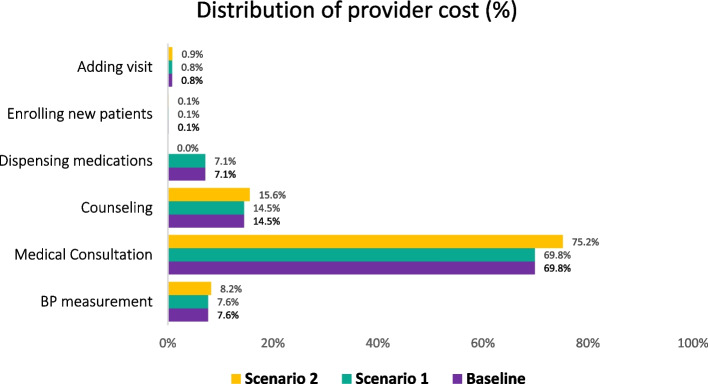
Distribution of provider cost by activity

Figure 5 explores the relative contribution of different provider activities to per-patient provider costs. This distribution does not vary widely by scenario. Medical consultation had the highest share of provider costs (70–75%), followed by counseling (15%), with smaller contributions by the time spent on measuring BP, dispensing medications, and adding visits to the e-Registry.

## Discussion

This study examined the annual costs associated with delivering HTN services under the Healthy Hearts program at seven municipal RHUs in District 1 of Iloilo province. Three scenarios were assessed for medication and provider costs, depending on the procurement scheme of HTN medications – local procurement (baseline scenario), PPPI national pooled procurement (Scenario 1), and private pharmacy outsourcing (Scenario 2). A main driver of provider time costs came from serving patients with uncontrolled HTN, with an average annual provider cost of PhP 1,935 (USD 32.9) per patient for uncontrolled patients vs PhP 293 (USD 5) for controlled patients. This difference was driven by the higher cost of provider time spent on following up uncontrolled patients, who require more frequent follow-up visits. Medication costs, on the other hand, can vary considerably depending on the medication procurement mechanism. Average medication costs per patient per year were estimated at PhP 534 (USD 9.1) using local procurement prices (range PhP 423–675), PhP 170 (USD 2.9) using PPPI pooled procurement prices (range PhP 133–217), and PhP 1353 (USD 23) using private pharmacy outsourced prices (range PhP 1,052–1,798).

The outsourcing scenario for medication procurement was applied to inform the viability of using private pharmacies for supplying protocol medicines as this supply becomes decentralized to the local government units. Outsourcing could potentially bolster local medicine supply by leveraging the local pharmacies’ existing supply chain and relationship with pharmaceutical suppliers. However, our results suggest that currently private pharmacy outsourcing is more expensive than other alternatives for medication procurement, and that higher medicine costs in the outsourcing scenario would be only slightly offset by lower provider costs that would occur in the event of outsourcing the dispensing of medications to external pharmacies. In the outsourcing scenario, providers working in the RHU no longer spend time dispensing medication to patients, reducing program labor costs by up to PhP 81 (USD 1.4) per patient per year. Taken together, these findings suggest that an ideal scenario for cost control would result from combining the pharmacy outsourcing model with pharmacies procuring medications through the national PPPI pool.

Pooling the procurement of medicines to higher levels of the health system where economies of volume can be achieved can help to address the challenge of high medication costs. Dubois et al. [14] analyzed procurement drug prices from seven low- and middle-income countries with diverse drug procurement systems, including the Philippines. They concluded that centralized procurement systems allow public buyers to obtain significantly lower prices of essential medicines.

The pooled procurement prices for amlodipine and losartan used for Scenario 1 in our study were those the DOH could obtain centrally from PPPI through annual purchase orders, plus 8% to cover logistics costs. In order for municipal LGUs to approximate these prices, they would need to explore mechanisms to pool their funds at a regional or province level. One potential mechanism is the ‘special health fund’ which province-wide health systems are mandated to establish under the 2019 UHC Act to “pool and manage all resources intended for health services to finance population-based and individual-based health services, health system operating costs, capital investments, and remuneration of additional health workers and incentives for all health workers” [2].

The 2019 Philippines UHC Act was enacted to guarantee equitable access to quality and affordable health care for all Filipinos, protecting them from financial risks. To kickstart a comprehensive approach to primary care, PhilHealth introduced the Philhealth Konsultasyong Sulit at Tama (Konsulta) Package. All persons registered with an accredited PhilHealth Konsulta Provider are eligible for non-communicable disease (NCD) screening and risk assessment, health education, medical consultations, and selected laboratory and diagnostic tests and medicines for the management of diseases such as hypertension. To be accredited as a PhilHealth Konsulta Provider, local Rural Health Units (RHUs) need to meet requirements for pharmacy services alongside other standards [15]. Once accredited, RHUs are eligible to receive a capitation amount for delivering the Konsulta package of PhP 500 (USD 8.5) per person per year, with remaining expenditures contributed by LGUs. Thus, when medication costs exceed PhilHealth's capitation, LGUs would need to co-finance to ensure that patients get necessary prescriptions without financial strain. Innovations to reduce prices and strengthen supply chains, such as pooling medication procurement, will directly benefit LGUs and enhance healthcare sustainability.

This study has several limitations. First, it was restricted to seven RHUs in District 1 of Iloilo province; thus, the findings may not be easily generalizable to the province or national level. The selection of the RHUs and study participants may reflect some degree of selection bias as well as reporting bias. There are intangible savings that could be associated with the pharmacy outsourcing model that are not accounted for here. These include improvements in medicine access (as pharmacies are open at night and on weekends) and freeing up storage space at RHUs. Since the outsourcing model was limited in terms of time and scope during the Healthy Hearts demonstration project, there was little incentive for pharmacies to provide competitive prices. An outsourcing scenario that is employed on a larger scale might result in more advantageous medicine prices for participating RHUs than the prices explored here. Finally, provider time costs and medicine costs are mutually exclusive, so that the marginal impacts of variations in these costs on the overall cost are independent of each other. The cost data were collected and validated using the combination of key informant interviews (KII), focus group discussions (FGD), and secondary data sources to estimate and validate various cost inputs, associated parameters, and assumptions.

## Conclusions

The findings from this study indicate two key areas for potential cost savings when implementing hypertension management programs in the Philippines. One is enhancing the control of HTN, such as through employing standardized treatment approaches, thereby reducing the need for follow-up visits and cutting down on provider time costs. Provider time costs can also be partially reduced through outsourcing the dispensing of medications to private pharmacies, although doing so would result in steep rise in medication costs at current private pharmacy prices.

We find that mechanisms for pooled medication procurement, such as the PPPI, can address the challenge of high medication costs. Despite these efforts, certain trends may continue to contribute to the escalation of healthcare costs. These include the growing demand for healthcare services due to population increase, aging, comorbidities, changes in lifestyle, and poor patient adherence to medications. A multifaceted strategy that encompasses patient education, better medication adherence, and innovative service delivery and procurement methods may be necessary to address these concerns.

## Supplementary Information

Supplementary Material 1.

## Data Availability

All data used in this study is available as a supplementary file.
